# Revertant mosaicism repairs skin lesions in a patient with keratitis-ichthyosis-deafness syndrome by second-site mutations in connexin 26

**DOI:** 10.1093/hmg/ddx017

**Published:** 2017-02-01

**Authors:** Sanna Gudmundsson, Maria Wilbe, Sara Ekvall, Adam Ameur, Nicola Cahill, Ludmil B. Alexandrov, Marie Virtanen, Maritta Hellström Pigg, Anders Vahlquist, Hans Törmä, Marie-Louise Bondeson

**Affiliations:** 1 Department of Immunology, Genetics and Pathology, Science for Life Laboratory, Uppsala University, Uppsala, Sweden; 2 Theoretical Biology and Biophysics (T-6), Los Alamos National Laboratory, Los Alamos, NM, USA and; 3 Department of Medical Sciences, Dermatology, Uppsala University, Uppsala, Sweden

## Abstract

Revertant mosaicism (RM) is a naturally occurring phenomenon where the pathogenic effect of a germline mutation is corrected by a second somatic event. Development of healthy-looking skin due to RM has been observed in patients with various inherited skin disorders, but not in connexin-related disease. We aimed to clarify the underlying molecular mechanisms of suspected RM in the skin of a patient with keratitis-ichthyosis-deafness (KID) syndrome. The patient was diagnosed with KID syndrome due to characteristic skin lesions, hearing deficiency and keratitis. Investigation of *GJB2* encoding connexin (Cx) 26 revealed heterozygosity for the recurrent *de novo* germline mutation, c.148G > A, p.Asp50Asn. At age 20, the patient developed spots of healthy-looking skin that grew in size and number within widespread erythrokeratodermic lesions. Ultra-deep sequencing of two healthy-looking skin biopsies identified five somatic nonsynonymous mutations, independently present *in cis* with the p.Asp50Asn mutation. Functional studies of Cx26 in HeLa cells revealed co-expression of Cx26-Asp50Asn and wild-type Cx26 in gap junction channel plaques. However, Cx26-Asp50Asn with the second-site mutations identified in the patient displayed no formation of gap junction channel plaques. We argue that the second-site mutations independently inhibit Cx26-Asp50Asn expression in gap junction channels, reverting the dominant negative effect of the p.Asp50Asn mutation. To our knowledge, this is the first time RM has been reported to result in the development of healthy-looking skin in a patient with KID syndrome.

## Introduction

Keratitis-ichthyosis-deafness (KID) syndrome (OMIM #148210) is a rare congenital ectodermal disorder characterized by keratitis, sensorineural hearing loss, and ichthyosis/erythrokeratoderma that manifests as patches of red, thickened, scaly, and dry skin (1). About 15% of KID syndrome patients are reported to develop squamous cell carcinoma (SCC) of the skin and oral mucosa (2). The gap junction beta 2 (*GJB2;* NM_004004.5) gene of 2250 base pairs (bp) comprises two exons of which one exon, exon two, encodes the short 226 amino acid protein connexin (Cx) 26. To date, nine mutations (3) in Cx26 have been reported to cause KID syndrome in altogether approximately 100 cases (4). The dominant mutation *GJB2* c.148G > A, p.Asp50Asn is the most common one (5–9). The diseases-phenotype has been demonstrated in a mouse model expressing the *GJB2* c.50C > T, p.Ser17Phe mutation, a mutation reported to cause KID syndrome in humans (10). Mutations in *GJB2* are also associated with a number of other diseases, including Vohwinkel syndrome (OMIM #124500), Bart–Pumphrey syndrome (OMIM #149200), syndromic sensorineural hearing loss with keratoderma (OMIM #148350), hystrix-like ichthyosis-deafness syndrome (OMIM #602540), and nonsyndromic deafness (OMIM #601544, #220290).

**Figure 1:**
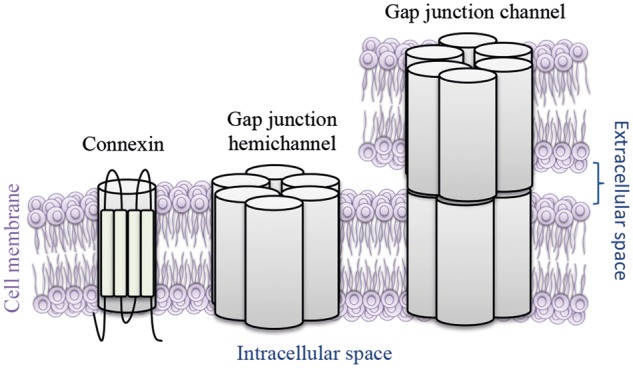
Schematic overview of gap junction channels. Gap junction channels connect the cytoplasm of two neighboring cells by transmembrane gap junction hemichannels that are comprised of six connexin subunits.

The Cx26 protein is a member of the Cx family that is comprised of structurally related transmembrane proteins that assemble to form gap junction channels (GJC) (11). Each GJC is composed of two hemichannels, which themselves are composed of six Cx protein subunits (Fig. 1). The *GJB2* gene is expressed in various tissues throughout the body but Cx26 has specifically been demonstrated to be co-expressed with other Cx proteins (Cx30, Cx31 and Cx43) in human epidermal keratinocytes and cochlea (4,12,13), i.e. tissues where the KID phenotype is expressed. The permeability of mutant GJCs has been functionally investigated, but the results of such studies are contradictory. The experiments resulted in reduced translocation, increased cell death, increased membrane flow, lowered levels and slower diffusion of molecules (14), and hyperactive hemichannels (15). Thus, even though no univocal mechanisms for KID syndrome have been found all studies conclude that KID syndrome likely is caused by the altered GJC permeability due to Cx mutations.

Revertant mosaicism (RM) is the result of spontaneous correction of a pathogenic mutation in a somatic cell including back mutation, gene conversion, intragenic recombination, and second-site mutation. Thus, RM leads to reversion of a genetic disease by correcting the disease-causing mutation or by introducing a novel mutation that inhibits the disease-causing mechanism (16). RM was first described in a patient with Lesch–Nyhan syndrome (17) and further illuminated by Jonkman in the genetic skin disorder epidermolysis bullosa (18). Since then, RM has been described in several diseases (19) and has recurrently been proposed as a possible model for skin therapy. It is suggested that reverted cells that give rise to healthy skin have a positive selective advantage because of normalized function, which could be of use in gene therapy (16). Transplanting reverted cells has had promising results (20), but implementation in routine treatment has not been successful (21). However, transplantation of endogenous revertant skin patches to affected areas has shown good results (22). To date, there is one report linking RM to KID, where a fetus expressed a lethal KID syndrome mutation (p.Gly45Glu) due to reversion of a protective nonsense mutation, which prevented expression of the mutation in the mother (23).

Herein, we elucidate the molecular mechanism giving rise to the healthy-looking phenotype by using single molecule real-time (SMRT) deep sequencing, which allow detection of second-site mutations present on the same allele as the disease-causing mutation by long sequencing reads. Ultra-deep sequencing was performed on genomic DNA and cDNA from healthy-looking and affected tissue, followed by investigation of the novel somatic mutations (SMs) molecular effect on GJC formation in HeLa cells.

## Results

### Case report

**Figure 2:**
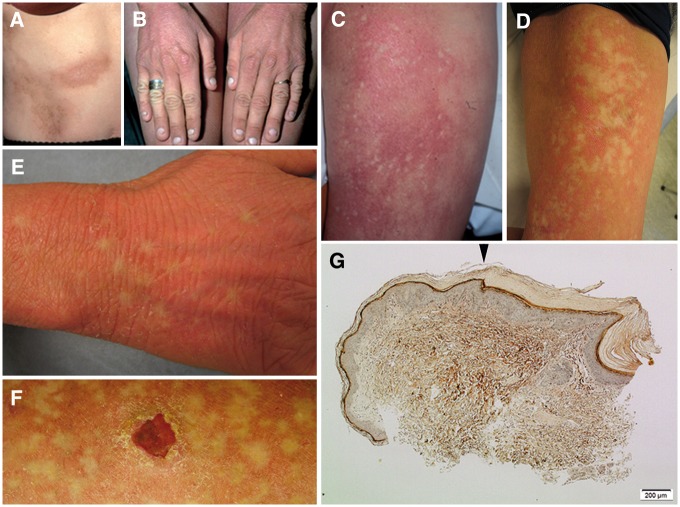
Images of skin lesions, healthy-looking spots, and epidermal histology. (**A,B**) The patients characteristic skin lesions, present since early childhood. (**C**) Intense erythema was present bilaterally on the thighs, and a few small spots of bleached skin within the erythema were observed at age of 20. (**D,E**) The non-inflamed areas of normal skin expanded and spread over time. (**F**) She also developed squamous cell carcinoma (SCC) at age of 30. (**G**) A skin biopsy of the edge between lesional and healthy-looking skin showed a relatively sharp shift (arrowhead) in epidermal histology from ortho-keratotic hyperkeratosis, hypergranulosis, and slight acanthosis (right) to almost healthy-looking skin (left). Image obtained using Masson’s staining: original magnification 100x.

The female patient, born in 1976, had experienced skin lesions, hearing impairment and keratitis typical of KID syndrome since early childhood (Fig. 2A and B). She was later diagnosed with a *de novo* recurrent *GBJ2* mutation, c.148G > A, p.Asp50Asn (5). At age 20 a few new mm-sized, punched-out spots of healthy-looking skin developed within the erythema on the inside of her thighs (Fig. 2C) that, with time, grew in size and number (Fig. 2D). At the age of 26, slowly expanding spots of healthy-looking skin also appeared on the back of her hands (Fig. 2E). A skin biopsy of the area between lesional and healthy-looking skin showed a clear-cut shift in epidermal histology from typical characteristics of KID syndrome to almost healthy-looking skin (Fig. 2G). At 30 years of age, she developed invasive SCC on her left thigh (Fig. 2F), which was surgically removed with no signs of spreading. The patient’s skin problems have been exaggerated by hidradenitis suppurativa in the axillae and groin, requiring long-term antibiotics, oral retinoids and CO_2_ laser surgery. After informed consent was obtained from the patient, punch biopsies were collected from lesional and healthy-looking spots on both of the inner aspects of her thighs.

### Ultra-deep sequencing identified SMs in *GJB2in cis* with the p.Asp50Asn mutation causing KID syndrome

To investigate the underlying molecular mechanisms SMRT deep sequencing of *GJB2* was performed on cDNA and DNA from two skin biopsies of healthy-looking spots and cDNA from lesional skin using the Pacific Biosciences RSII System. This generated more than 10,000 sequence reads of the *GJB2* locus in all of the samples. A total of five novel nonsynonymous SMs were identified: c.136G > A; p.Asp46Asn and c.443C > A; p.Ala148Asp in biopsy one and c.61G > A; p.Gly21Arg, c.137A > C; p.Asp46Ala and c.413G > A; p.Ser138Asn in biopsy two (Table 1). The KID syndrome-causing mutation *GJB2* c.148G > A; p.Asp50Asn and a common homozygous polymorphism outside the coding exon at position Chr13:20762956 (rs3751385) were also identified in both biopsies. By using SMRT sequencing technology, generating 1033 bp reads, all five novel SMs were demonstrated to be independently located *in cis* with the mutation causing KID syndrome. The same SMs were identified at both DNA and cDNA level at frequencies of 2.4–12.5% (Table 2). No SMs could be detected on the wild-type (wt) *GJB2* allele in the biopsies of healthy-looking skin or on any alleles from the biopsies of lesional tissue.

**Table 1:** Bioinformatic predictions of the five somatic mutations identified with single molecule real-time sequencing

Protein	cDNA	Genomic (GRCh37/hg19)	MutationTaster	PhyloP	SIFT	Reported
p.Gly21Arg	c.61G>A	Chr13:20763660	disease-causing	highly conserved (5.94)	deleterious 0	Rabionet et al. 2006
p.Asp46Asn	c.136G>A	Chr13:20763585	disease-causing	highly conserved (5.94)	deleterious 0	Bazazzadegan et al. 2011
p.Asp46Ala	c.137A>C	Chr13:20763584	disease-causing	highly conserved (4.89)	deleterious 0	This report
p.Ser138Asn	c.413G>A	Chr13:20763308	polymorphism	weakly conserved (1.09)	tolerated (0.2)	Snoeckx et al. 2005
p.Ala148Asp	c.443C>A	Chr13:20763278	disease-causing	moderately conserved (2.38)	deleterious 0.01	This report

Five somatic mutations were identified in two affected skin biopsies. Bioinformatic tools predicted the variants to be disease-causing, conserved and deleterious suggesting and the variant might have an effect on the protein (except p.Ser138Asn, predicted to be a polymorphism, weakly conserved and tolerated).

**Table 2:** Single molecule real time sequencing of two skin biopsies from healthy-looking spots

Frequency of reads %			Variants detected			
Biopsy one	gDNA	cDNA	c.136G>A	c.148G>A	c.443C>A	
Read one	46.5	55.3	wt	wt	wt	
Read two	31.3	26.5	wt	**mut**	wt	
Read three	9.8	12.5	**mut**	**mut**	wt	
Read four	7.8	4.2	wt	**mut**	**mut**	

Biopsy two	gDNA	cDNA	c.61G>A	c.137A>C	c.148G>A	c.413G>A

Read one	48.7	53.0	wt	wt	wt	wt
Read two	34.5	21.0	wt	wt	**mut**	wt
Read three	4.7	11.8	wt	**mut**	**mut**	wt
Read four	6.9	7.6	**mut**	wt	**mut**	wt
Read five	2.4	5.4	wt	wt	**mut**	**mut**

Read one represents the wild-type allele, read two represent the c.148G > A; p.Asp50Asn allele without somatic mutations (SM) and read three-five represent the c.148G > A; p.Asp50Asn mutant alleles with SMs. The wt*GJB2* allele was detected in approximately 50% of the reads and was not affected with second-site SMs. The c.148G > A; p.Asp50Asn *GJB2* allele was detected in 31.3% (gDNA) and 26.5% (cDNA) of all reads in biopsy one, 34.5% (gDNA) and 21.0% (cDNA) of all reads in biopsy two. The five novel second-site SMs were identified on the c.148G > A; p.Asp50Asn allele solely at frequencies of 7.8–9.8% (gDNA) and 4.2–12.5% (cDNA) in biopsy one, 2.4–6.9% (gDNA) and 5.4–11.8% (cDNA) in biopsy two.

### No expression of Cx26-Asp50Asn with SMs in GJC plaques in HeLa cells

**Figure 3:**
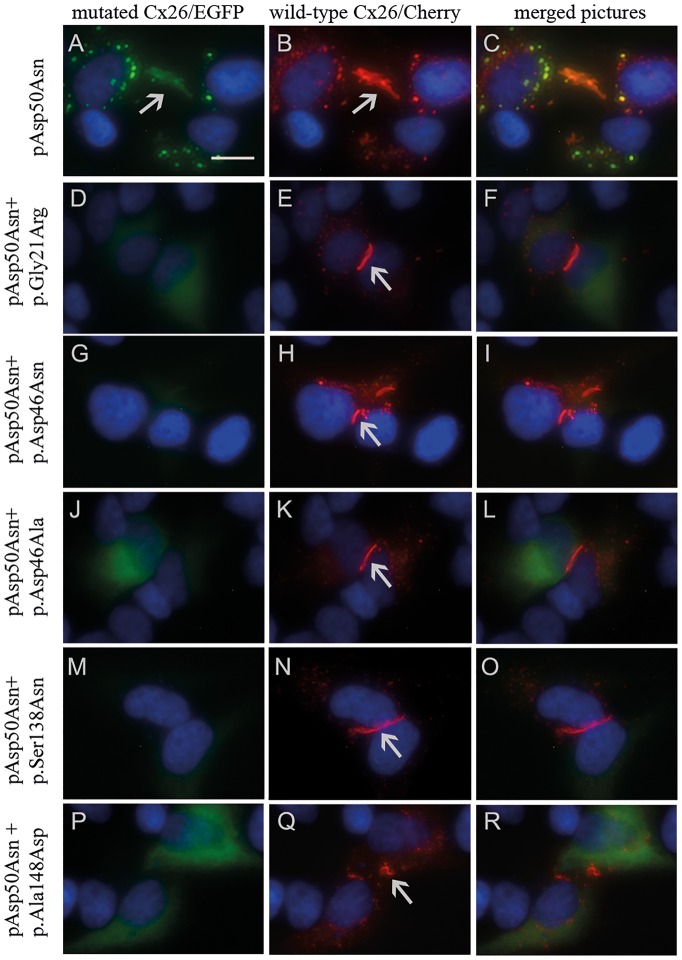
Transfection results displaying wtCx26, Cx26-Asp50Asn, and Cx26-Asp50Asn expressing all somatic mutations (SMs) individually. Arrows mark gap junction channel (GJC) formation. (**A–C**) Display formation of similar GJCs in Cx26-Asp50Asn marked with green fluorescent protein and wtCx26 marked with red fluorescent protein. The scale bar is 15 µm. (**D–R**) Display formation of GJCs when expressing wtCx26 marked with red fluorescent protein, but not when expressing Cx26-Asp50Asn with additional SMs labeled with green fluorescent protein.

The five novel SMs’ effect on Cx26 with KID syndrome-causing mutation p.Asp50Asn (Cx26-Asp50Asn) was investigated by *in vitro* studies of the protein expression in GJC. Transfection experiments were performed in HeLa cells that did not express endogenous Cx26 (15). Co-transfection was performed with wtCx26 fused to enhanced green fluorescent protein (EGFP) and Cx26-Asp50Asn or Cx26-Asp50Asn with one of the five SMs fused to the red fluorescent protein mCherry. Cx26-Asp50Asn formed GJC plaques in the same manner as wtCx26 (Fig. 3A–C). In contrast, Cx26-Asp50Asn with SMs did not form GJC plaques. However, a weak, evenly distributed fluorescent signal could be detected inside the cytoplasm (Fig. 3D, G, J, M and P).

### Proximity ligation-based western blot assay confirms intracellular expression of Cx26

**Figure 4:**
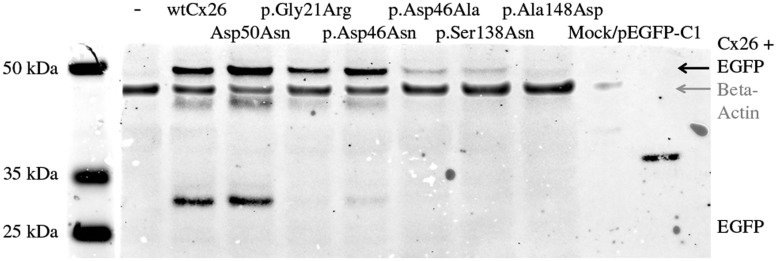
Protein extraction and results of the PLA-WB assay. The assay reveals expression of Cx26/EGFP protein in HeLa cells transfected with p.Gly21Arg, p.Asp46Asn (high expression), p.Asp46Ala, p.Ser138Asn, and p.Ala148Asp (low expression), the band size corresponding to the size of wtCx26 and Cx26-Asp50Asn (about 53 kDa). Beta-actin (42 kDa) was used as a positive control for protein extraction.

Intracellular expression of Cx26-Asp50Asn with secondary SMs (Fig. 3D, G, J, M and P) was investigated using a proximity ligation-based western blot (PLA-WB) assay. Primary antibodies targeting GFP detected proteins of about 53 kDa (EGFP 26 kDa plus Cx26 27 kDa) in all samples (Fig. 4), confirming the intracellular expression of Cx26 in HeLa cells.

## Discussion

This study is the first one, to our knowledge, to describe RM that resulted in the development of healthy-looking skin in a patient with KID syndrome. Two biopsies from healthy-looking tissue, including the epidermis (consisting of >95% keratinocytes) and superficial dermis (mostly consisting of extracellular matrix and, to a less extent, fibrocytes and inflammatory cells), were investigated with ultra-deep sequencing. Five novel missense SMs were found to be independently present *in cis* with the disease-causing mutation *GJB2* c.148G > A, p.Asp50Asn. Three of the five mutations, p.Gly21Arg (24), p.Asp46Asn (25) and p.Ser138Asn (26) have previously been reported in hearing loss. The mutations p.Asp46Ala and p.Ala148Asp, have not been reported previously, however, two other mutations, p.Asp46Asn and p.Asp46Glu, have been described in the same codon as p.Asp46. Both of the previously unreported mutations, p.Asp46Ala and p.Ala148Asp, are classified as “likely pathogenic” since they have not been reported in the normal population in the Exome Aggregation Consortium (ExAC) (27) and have been predicted to be “disease-causing” by bioinformatics tools (Table 1).

To elucidate the molecular mechanism driving the mutational process in the patient we investigated the presence of signature patterns. Endogenous and exogenous mutational processes (e.g. smoking, exposure to ultraviolet [UV] light, or a primary driver mutation) leave a unique molecular pattern of SMs in the genome of exposed cells, called a mutational signature (28). A large-scale analysis of thousands of cancer patients revealed 30 distinct mutational signatures, some of known etiologies (29,30). Quantification of the similarity between the SMs identified in this study, and known mutational signatures,reveals that exposure to UV light is the most likely cause.

Epidermal keratinocytes are situated close to the skin surface and are naturally exposed to UV-light. Therefore, they are at increased risk of SMs, some of which may potentially result in a nullifying of dominant-negative effects of the mutant protein. Also, one can hypothesize that KID syndrome patients have an enhanced risk of UV-induced mutations due to inflamed skin. Additionally, it is interesting to note that in our study, SCC occurred in the same location as RM. Very few SMs are known and therefore one can only speculate about the possible mutation patterns.

Functional studies of HeLa cells demonstrated that Cx26-Asp50Asn with SMs did not form GJC plaques (Fig. 3D, G, J, M and P), in contrast to wtCx26 and Cx26-Asp50Asn, which formed similar and distinct GJC plaques (Fig. 3A–C). Considering the results of previous studies (9,14,15,31) and our results, we suggest that independent introduction of p.Gly21Arg, p.Asp46Asn, p.Asp46Ala, p.Ser138Asn, or p.Ala148Asp *in cis* with the p.Asp50Asn mutation inhibits the dominant negative effect of the p.Asp50Asn mutation. This hypothesis is strengthened by the fact that SMs only could be detected on the p.Asp50Asn-mutated allele.

It is likely that nullifying SMs randomly also occur on the wtCx26 allele of the patient, although in this case leading to cell death because of detrimental suppression of functional wtCx26. In contrast, keratinocytes expressing SMs *in cis* with the Cx26-Asp50Asn mutation undergo positive selection as an expression of a higher proportion of wtCx26 contributes to the normalized molecular diffusion. This restores the epidermal barrier (as revealed in the histological investigation, Fig. 2G), leading to reduced skin inflammation and alleviation of the ichthyosis observed in the patient’s lesional skin. The intracellular fluorescent signal (Fig. 3D, G, J, M and P) and the PLA-WB assay of Cx26-Asp50Asn with SMs (Fig. 4) indicate that Cx26-Asp50Asn with SMs is expressed within the HeLa cells. Hence, we suggest that Cx26-Asp50Asn with secondary SMs are translated but could suggestively be intracellularly detained during post-translational processes, e.g. during oligomerization to hexameric units within the endoplasmic reticulum (32,33).

Conclusively, we report a KID patient with RM causing the development of healthy-looking skin. SMRT deep sequencing identified five second-site SMs *in cis* with the disease-causing mutation. We propose that reverted skin phenotype in our patient occurs because the SMs inhibit Cx26-Asp50Asn expression in GJCs, leading to reversion of the dominant negative effect of p.Asp50Asn. Increased knowledge of RM etiology leads to the understanding of biological processes that can be used in gene therapy of genetic diseases. Also, underlying molecular mechanisms of SMs should be further investigated since they can predispose cancer as well as other genetically complex diseases.

## Materials and Methods

### Ethical consent

The local ethics committee in Uppsala approved this study (Dnr 2012/523). Informed consent was obtained from the patient and all clinical investigation and genetic analyses were conducted in accordance with the guidelines of the Declaration of Helsinki.

### Histopathologic examination and preparation of DNA and cDNA from skin samples

The skin was infiltrated with lidocaine adrenaline and punch biopsies were obtained, fixed in formaldehyde and embedded in paraffin. Masson’s staining of tissue sections for microscopic analysis was performed according to standard protocol. Two biopsies from lesional skin and two biopsies from healthy-looking skin were placed in 1 ml RNAlater (Ambion, Foster City, CA). Total RNA was extracted with TriReagent (Ambion) and dissolved in 25 µl DEPC water. One µg total RNA was used for cDNA synthesis with oligo d(T) and 200 U M-MLV Reverse Transcriptase (Life Technologies, Carlsbad, CA). DNA was extracted from all biopsies using an EZ1 DNA investigator kit (Qiagen, Hilden, Germany).

### Single molecule real-time sequencing using the Pacific Biosciences RSII system

A 1033 bp *GJB2* fragment was amplified from cDNA and DNA according to standard Taq polymerase chain reaction (PCR) protocol (Applied Biosystems, Waltham, MA) at 95° 5’, for 20 cycles (95° 20’’, 65-55° 30’’, 72° 1’) and 25 cycles (95° 20’’, 55° 30’’, 72° 1’) with M13-tagged *GJB2* primers (Supplementary Material, Table S1). SMRTbell™ libraries were produced using the Pacific Biosciences 1.0 Template Preparation Kit according to manufacturer’s instructions (Pacific Biosciences, Menlo Park, CA). SMRTbells™ were constructed and sequenced according to recommended protocol for Pacific Biosciences 1 kb Template Preparation, on 400 ng DNA with the Pacific Biosciences RSII instrument using C3 chemistry and 180-min movie. Each SMRTbell™ amplicon library was loaded onto one SMRT cell and sequenced.

### Mutational analysis of pacific biosciences sequence data

Mutations in the *GJB2* gene were identified by aligning the Pacific Biosciences sequence reads to the *GJB2* reference sequence (hg19 assembly version) followed by variant calling using the Minor Variant Caller tool available in the SMRT analysis portal. A separate analysis was performed for each sample, revealing 6 mutations in the *GJB2* transcript at cDNA positions 136, 148, and 443 in biopsy one and at positions 61, 137, 148, and 413 in biopsy two (Table 2). For each sample, high-quality circular consensus (CCS) reads were constructed using the reads of insert plug-in in the SMRT analysis portal. CCS reads not perfectly matching the forward and reverse primer sequences were removed. Filtered CCS reads were then used as the input for CAVA tool (https://github.com/National Genomics Infra structure/CAVA; date last accessed January 20, 2017), a computational method for analyzing mutations in long-read amplicon sequence data. The CAVA analysis determined the allele frequencies and the clonal composition of the six previously identified mutations in all the sequenced samples.

### Prediction of mutation signatures

Mutation signatures were predicted by deriving a score for all five SMs and examining their patterns (29), their average mutational burdens, and their overall prevalence in human tissue (34). The score was derived after performing jackknife resampling (35) and was normalized based on the score for the highest mutational signature (i.e. signature 7).

### Molecular cloning and mutagenesis

Expression vectors were assembled by amplifying *GJB2* in 25 ng cDNA from lesional tissue with primers containing restriction sites (Supplementary Material, Table S1) using standard Taq PCR protocol (described above). The PCR product was cloned into the pEGFP-C1 vector expressing green fluorescent protein (EGFP; Clontech Laboratories, Mountain View, CA,) using restriction enzymes EcoRI and BamHI (Thermo Scientific, Foster City, CA). Colonies with vectors expressing wt*GJB2* and *GJB2* c.148G > A were identified with Sanger sequencing on a 3130XL ABI genetic analyzer using an ABI PRISM Big Dye Primer v3.0 Cycle Sequencing Ready Reaction Kit (Applied Biosystems) with primers covering the vectors’ multiple cloning sites (Supplementary Material, Table S1). The sequences were analyzed using CodonCode Aligner V.5.0.1 (CodonCode Corporation, Centerville, MA). Vectors were obtained using an E.Z.N.A.® Plasmid Maxi Kit (Omega Bio-Tek, Norcross, GA). Wt*GJB2* was cloned into pmCherry-C1 vector expressing red fluorescent protein (Clontech Laboratories, Mountain View, CA) to enable co-expression of mutant *GJB2* and wt*GJB2.* Mutagenesis was performed for all five SMs on pEGFP-C1 with *GJB2* c.148G > A, p.Asp50Asn using a QuickChange II Site-Directed Mutagenesis Kit (Agilent Technologies, Santa Clair, CA). The protocol was optimized as described by Wang and Malcolm (36). Verification of SMs were performed by Sanger sequencing (described above; Supplementary Material, Fig. S1A–G).

### Immunofluorescent co-transfection in HeLa cells

Co-transfection was performed with 100 ng wtCx26/pmCherry-C1 (100 ng/µl) and 100 ng Cx26-Asp50Asn/pEGFP-C1 or Cx26-Asp50Asn combined with one of the five SMs fused to EGFP (100 ng/µl) in a 24-well plate with 80,000 HeLa cells/well using the JetPEI transfection protocol (Polyplus-transfection, Illkirch, France). All combinations were performed in triplicate. Cells were stained using Vectashield mounting medium with DAPI (Vector Laboratories, Burlingame, CA) and observed at 24 h, 48 h (Fig. 3; Supplementary Material, Fig. S2), and 72 h post-transfection using Invitrogen™ EVOS™ FL 6 microscope (Thermo Fisher Scientific, Waltham, MA). Images were magnified 40x with optimized contrast and brightness to balance the detected fluorescence and allow qualitative analysis.

### Protein extraction and proximity ligation-based western blot assay

Intracellular expression of Cx26-Asp50Asn with SMs was investigated with PLA-WB on protein from HeLa cells transfected with 200 ng of each construct separately. For specific protocol, see Supplementary Material, Document S1.

## Supplementary Material

Supplementary Material is available at *HMG* online.

## Supplementary Material

Supplementary Data

Supplementary Figure S1

Supplementary Figure S2
