# Supplementary material for: Revertant mosaicism repairs skin lesions in a patient with keratitis-ichthyosis-deafness syndrome by second-site mutations in connexin 26
Source: Hum Mol Genet. 2017 Feb 1;26(6):1070–7. doi: 10.1093/hmg/ddx017 (PMC5409067; doi:10.1093/hmg/ddx017)
Supplement: Supplementary Data [file ddx017_supp.doc]

**Supplementary material**

**S1 Table. All primers used for PCR, Sanger sequencing, SMRT sequencing, and mutagenesis.**

| **Primers** | **Forward** | **Reverse** |
| --- | --- | --- |
| PCR and Sanger Sequencing of *GJB2* | TGT AAA ACG ACG GCC AGT GAA CAA ACA CTC CAC CAG CA | CAG GAA ACA GCT ATG ACC TGT GGG AGA TGG GGA AGT AG |
| Primer for SMRT sequencing | GGG GTG CGG TTA AAA GGC GCC ACG G | GCA ACC ATT TGA AAC CCC T |
| GJB2 with restriction site | GTA TGA ATT CAG ATT GGG GCA CGC TGC A | TGG GAA GTC AAA AAA GCC AGT TTA AGG ATC CGC AT |
| Screening of MCS pmCherry-C1 | CAC CAT CGT GGA ACA GTA CG | GGA GGT GTG GGA GGT TT |
| Screening of MCS pEGFP-C1 | ACA TGG TCC TGC TGG AGT TC | GGG AGG TGT GGG AGG TTT T |
| Mutagenesis G21R | GGT GAG CCA GAT CTT TCT AAT GCT GGT GGA GTG TT | AAC ACT CCA CCA GCA TTA GAA AGA TCT GGC TCA CC |
| Mutagenesis D46N | TTG GCC TGC TCA TTT CCC CAC ACC TCC | GGA GGT GTG GGG AAA TGA GCA GGC CAA |
| Mutagenesis D46A | GTT GGC CTG CTC AGC TCC CCA CAC CTC | GAG GCT CCC CAC ACT GAG CAG GCC AAC |
| Mutagenesis S138N | AAG AAG ATG CTG TTT GTG TAG GTC CAC CAC AGG GA | TCC CTG TGG TGG ACC TAC ACA AAC AGC ATC TTC TT |
| Mutagenesis A148D | GAC GTA CAT GAA GGC GTC TTC GAA GAT GAC CCG | CGG GTC ATC TTC GAA GAC GCC TTC ATG TAC GTC |

**S1 Figure. Sanger sequencing of vectors.** The mutation of interest is highlighted in black and indicated by a blue line. (A) Sanger sequencing of pmCherry-C1 vector expressing wtGJB2. (B) Sanger sequencing of pEGFP-C1 vector withholding disease-causing GJB2 c.148G>A mutation. Sanger sequencing of pEGFP-C1 confirmed expression of GJB2 with c.148G>A and all five SMs (C) c.61G>A, (D) c.136G>A, (E) c.137A>C, (F) c.413G>A and (G) c.443C>A independently.

**S2 Figure. Positive and negative controls of the transfection assay.** Arrows mark GJC formation. (A-B) The transfection revealed that wtCx26 labeled with green fluorescent molecules and wtCx26 labeled with red fluorescent molecules have similar expressions. The scale bar is 15 µm. (D-F) Display HeLa cells transfected with vectors expressing only the fluorescent molecules EGFP and pmCherry-C1. (G-I) There is no detectable background fluorescence from HeLa cells treated with only JetPEI transfection reagents.

**S1 Document. Specific protocol for WB-PLA assay.**

HeLa cells were transfected and incubated for 48 h as described above with 200 ng of each constructs separately (pEGFP-C1 vector, vector with wtCx26, Cx26-D50N, and Cx26-D50N expressing all five SMs individually). Protein was extracted according to manufactures protocol with 100 µl RIPA/well (Sigma, St. Louis, MO). A PLA-WB assay was performed on 13 ul protein lysate loaded in Invitrogen Novex® 12% Tris-glycine pre-cast gel (Thermo Fisher Scientific) and run at 150 V for 1 h using the miniVE Vertical Electrophoresis System (GE Healthcare, Little Chalfont, UK). The proteins were transferred to nitrocellulose membranes using the Blot® 2 Dry Blotting

System (Life Technologies), followed by incubation for 1 h at room temperature in a blocking solution (Odyssey™ Blocking Buffer (TBS), Licor). The membrane was then incubated overnight at 4 °C with (B-2) sc-9996 anti-GFP primary antibodies (Santa Cruz, Dallas, TX) in Odyssey™ Blocking Buffer at 1:200 dilution. The membrane was washed three times for 5 min in Tris-buffered saline with Tween (TBS-T; 0.05 M Tris base, 150 mM NaCl, pH 8.4, with 0.05% Tween 20), followed by incubation for 2 h at 37 °C with anti-mouse PLUS and anti-mouse MINUS PLA probes (Olink Bioscience, Uppsala, Sweden) in Odyssey™ Blocking Buffer, 1:5 dilution. The membrane was then washed three times for 5 min with TBS-T, followed by incubation in Duolink II Ligation solution (Olink Bioscience, Uppsala, Sweden) at 37 °C for 30 min and two additional washes in TBS-T for 2 min. The ligated DNA circle was amplified with Fermentas Phi29 Polymerase (Thermo Fisher Scientific, Waltham, MA), and single-stranded RCA products were visualized with an Alexa Fluor 700 hybridization probe (5'-CAG TGA ATG CGA GTC CGT CTU UUU-3'; Integrated DNA Technologies) in Fermentas Phi29 Polymerase Buffer (Thermo Fisher Scientific) and 2.5 mM dNTPs (Sigma-Aldrich). Washing was performed three times for 5 min in TBS-T. The membrane was then co-stained with sc-(I-19) 1616 rabbit anti-actin antibodies (Santa Cruz) detected using IRDye® 800CW Donkey anti-Rabbit IgG (Licor, Lincoln, NE) and imaged using an Odyssey Scanner (Licor).
