# Supplementary figures and images for: Revertant mosaicism repairs skin lesions in a patient with keratitis-ichthyosis-deafness syndrome by second-site mutations in connexin 26
Source: Hum Mol Genet. 2017 Feb 1;26(6):1070–7. doi: 10.1093/hmg/ddx017 (PMC5409067; doi:10.1093/hmg/ddx017)

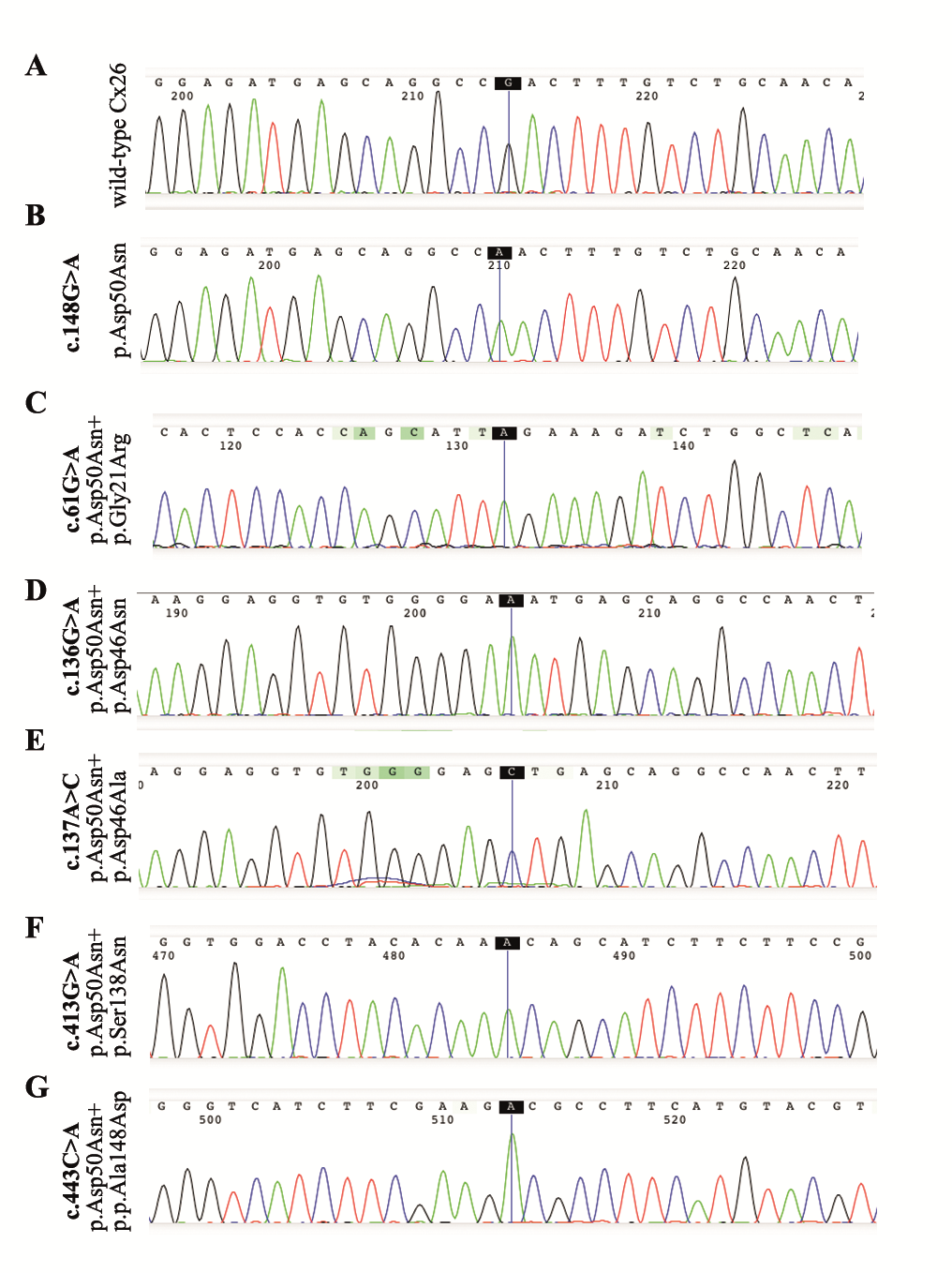

Supplement: Supplementary Figure S1 [file ddx017_supp_figs1.png]

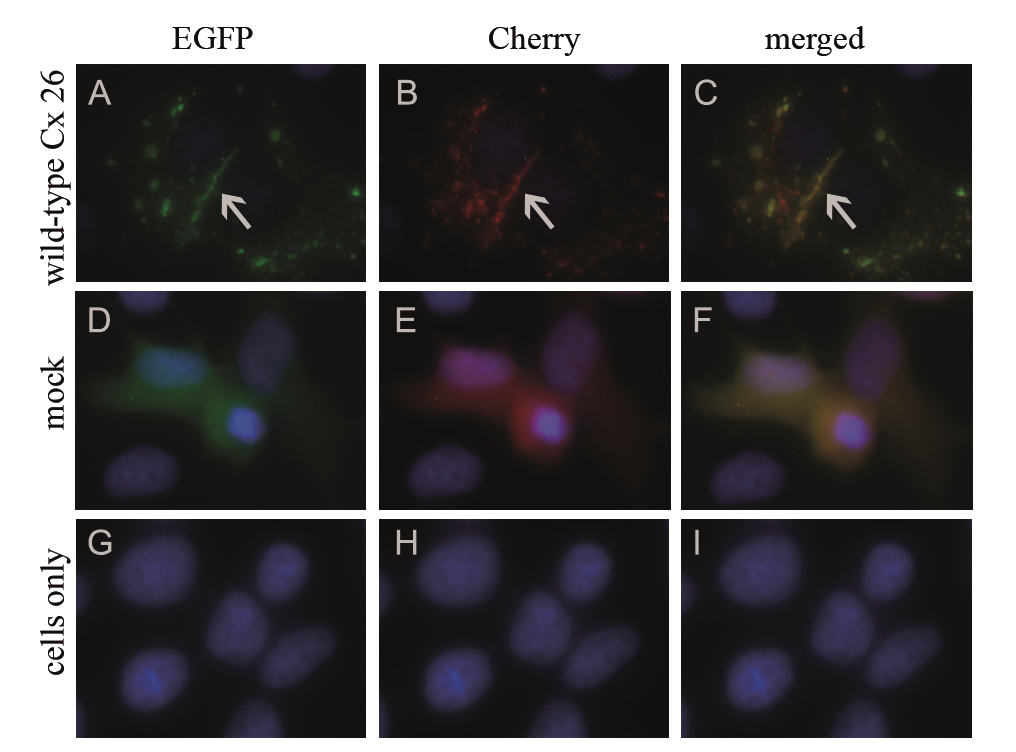

Supplement: Supplementary Figure S2 [file ddx017_supp_figs2.png]
